# How Are n-3 LCPUFAs Antiarrhythmic? A Reassessment of n-3 LCPUFAs in Cardiac Disease

**DOI:** 10.1155/2012/746709

**Published:** 2012-08-16

**Authors:** Andrew Ramadeen, Paul Dorian

**Affiliations:** ^1^Keenan Research Centre, Li Ka Shing Knowledge Institute, St. Michael's Hospital, Toronto, ON, Canada M5B 1W8; ^2^Division of Cardiology, St. Michael's Hospital, Toronto, ON, Canada M5B 1W8; ^3^Department of Medicine, University of Toronto, Toronto, ON, Canada M5S 1A8

## Abstract

Long-chain n-3-polyunsaturated fatty acids (n-3 LCPUFAs), referring particularly to marine-derived eicosapentaenoic acid (EPA) and docosahexaenoic acid (DHA), have been shown to be effective in treating arrhythmias in some clinical trials and animal studies. The mechanism for this effect of n-3 LCPUFAs is not well understood. Experimental studies and clinical trials published in the 1980s and 1990s suggested that n-3 LCPUFAs may be antiarrhythmic drugs, but more recent trials have not confirmed this. In this paper, we examine evidence for, and against, the direct antiarrhythmic action of n-3 LCPUFAs and suggest that antistructural remodeling effects of n-3 LCPUFAs may be more relevant in accounting for their clinical effects.

## 1. Introduction

The putative benefits of n-3-polyunsaturated fatty acids (n-3 PUFAs) have been popularized in recent years. N-3 PUFAs have been applied to a diverse range of health concerns from antiaging treatments, to attenuating violent behavior in prison inmates, to improving infant intelligence [1–3]. The area of cardiovascular disease (CVD) has received a great deal of attention from n-3 PUFA researchers. Studies testing eicosapentaenoic acid (EPA) and docosahexaenoic acid (DHA), the long-chain n-3 PUFAs (n-3 LCPUFAs) obtained primarily from marine sources (algae, and fatty cold water fish like salmon, mackerel and tuna), to prevent and treat CVD have increased exponentially in recent years. These studies, both observational and randomized controlled trials (RCTs), have led to current American Heart Association guidelines which recommend eating fish high in n-3 LCPUFAs at least twice a week (http://www.heart.org/).

In the 1970s, Dyerberg and Bang published a series of studies showing that Greenland “Eskimos”, despite surviving on a diet containing a great deal of whale, seal, and fish fat, had virtually no incidence of CVD. This was in contrast to observations in neighboring Danish populations which had much higher incidence of CVD, but similar fat intakes (albeit from nonmarine sources). This led to the suggestion that the low CVD rates among Eskimos were the result of high intake of n-3 LCPUFAs and their putative antiatherogenic/antithrombotic effects [4]. In the 30 years since, n-3 LCPUFAs have been shown to have anti-inflammatory and antiplatelet properties, as well as the ability to lower blood pressure and triglycerides [5]. 

## 2. Clinical Studies of n-3 LCPUFAs and Arrhythmias

In 1989, results from the Diet And Reinfarction Trial showed that increasing fish intake significantly reduced all-cause mortality (by 29%) in 2,033 postmyocardial infarction (MI) men (see Table 1) [6]. The authors hypothesized that fish-mediated prevention of ventricular fibrillation (VF) could explain their results. A great deal of research followed to test the antiarrhythmic potential of n-3 LCPUFAs. In the 1990s, 2 large studies showed a benefit of n-3 LCPUFAs in preventing sudden death. The Physicians' Health Study observational trial followed ~20,000 men over 11 years and found that increasing fish intake was associated with a reduction in sudden cardiac death (SCD) presumably from VF (relative risk of SCD 0.48 for those eating fish at least 1 time/week) (see Table 2) [7]. The landmark GISSI Prevenzione Trial of ~11,000 post-MI patients found that n-3 LCPUFAs significantly reduced the risk of SCD, which became evident after only 4 months of followup (relative risk of SCD 0.47 in the n-3 LCPUFA group compared to controls) [8]. This clinical research was supported by basic science findings first in rats, and later in dogs and cell culture, showing that n-3 LCPUFAs can alter the electrophysiological properties of cardiac cells perhaps by affecting membrane fluidity or ion channel function [9, 10]. Thus there arose a perception that n-3 LCPUFAs may be a sort of “antiarrhythmic drug.”

However, more recent research is not entirely consistent with the idea that n-3 LCPUFAs are directly antiarrhythmic. Several observational studies and RCTs published in the last decade have not found any benefit of n-3 LCPUFAs in preventing arrhythmias. The 2010 Alpha-Omega RCT enrolled patients who were ~4 years post-MI, and no benefit of n-3 LCPUFAs was seen on any arrhythmic or other cardiac endpoint [11]. In the study nearly 5,000 patients took margarine enriched with EPA+DHA and the relative risk of a major cardiovascular event was 1.01. The 2010 OMEGA trial (not to be confused with Alpha-Omega) looked at ~4,000 patients who were 3–14 days post-MI and tested the ability of n-3 LCPUFAs on top of “modern therapy” to prevent SCD. After 1 year of followup, the results did not show any benefit of n-3 LCPUFAs on SCD or arrhythmia prevention [12]. Three recent randomized trials involving patients with ventricular arrhythmias and implantable cardioverter defibrillators (ICDs) also showed no benefit of n-3 LCPUFAs; one even showed a proarrhythmic trend with n-3 LCPUFAs [13]. Over 1 year of follow-up the relative risk of tachyarrhythmia or death in the n-3 LCPUFA groups was 0.72, 1.26, and 0.86 for a combined nonsignificant relative risk of 0.90. A recent meta-analysis of trials testing n-3 LCPUFAs for secondary prevention did not find any evidence to support a protective effect of n-3 LCPUFAs [14].

Trials to prevent atrial fibrillation (AF) have been equally disappointing. A 2010 randomized trial by Kowey et al. tested the ability of n-3 LCPUFAs to prevent recurrence of paroxysmal AF after cardioversion. There was no discernible effect of n-3 LCPUFAs for any measured outcome despite the use of very high doses (4–8 g/day) [15]. Among observational trials, data from the Framingham, Women's Health Initiative and Rotterdam studies show no effect of n-3 LCPUFAs on AF [16–18]. The 2010 Framingham Heart Study looked at ~4,500 members of the original and offspring Framingham cohorts and found no significant association between fish consumption and AF (relative risk 1.18 for highest quartile of fish consumption) [16]. Similarly, the 2010 Women's Health Initiative study recruited almost 45,000 postmenopausal women and also found no association between fish intake and incident AF (odds ratio 1.17 for highest quartile of fish intake) [17]. The 2006 Rotterdam study looked at 5,184 subjects and, again, no association was found between fish intake and AF (relative risk 1.18 for the highest tertile of fish intake) [18].

Several recent basic science studies have also failed to show a benefit of n-3 LCPUFAs in arrhythmia models. In a dog model of rapid atrial pacing leading to AF, n-3 LCPUFAs failed to reduce AF duration (duration with n-3 LCPUFAs 95% of untreated dog duration, P = NS) [19]. In a well-validated dog model of ischemia post-MI, dogs which were found to be vulnerable to ischemia-induced arrhythmias did not experience a reduction in VF incidence when treated with n-3 LCPUFAs (P = NS treated versus untreated). Interestingly, those dogs which were not found to be vulnerable to ischemia-induced arrhythmias post-MI suffered an increase in VF incidence when treated with n-3 LCPUFAs (*P* < 0.05 treated versus untreated) [20].

## 3. n-3 LCPUFAs as Antistructural Remodeling Agents

How can we reconcile the results of these recent negative studies with the positive results obtained from previous trials? It may be possible that n-3 LCPUFAs are not directly antiarrhythmic, but have other effects which indirectly lead to arrhythmia suppression. Structural remodeling of cardiac tissue is “arrhythmogenic,” and there is considerable evidence that n-3 LCPUFAs have anti-structural remodeling properties [19, 21]. If we consider n-3 LCPUFAs as agents which prevent the cellular response to injury (inflammation, apoptosis, hypertrophy, and fibrosis) which eventually lead to arrhythmias, instead of acting directly on cardiac electrical function as “antiarrhythmic” drugs, we may be able to resolve some of the seemingly discrepant results. This would position n-3 LCPUFAs as a kind of “upstream” therapy. The design of many negative trials testing the effect of n-3 LCPUFAs on arrhythmias did not allow them to detect potential structural-remodeling-related effects. In the case of the studies mentioned above, n-3 LCPUFAs were tested in disease states without a significant, active remodeling component, or in patients with very low event rates (as a result of aggressive modern therapy), or did not follow patients for long enough to observe a large number of events.

The anti-structural remodeling properties of n-3 LCPUFAs have been reported by multiple groups using multiple animal models of CVD. In a mouse aortic constriction model, n-3 LCPUFAs attenuated ventricular fibrosis and dysfunction [22]. Similarly, in a rat aortic-banding model, n-3 LCPUFAs were shown to prevent inflammation and dilatation of ventricular tissue [23]. In a sheep heart failure model (chronic intracoronary doxorubicin infusions), n-3 LCPUFAs reduced atrial enlargement, fibrosis, and AF duration [24]. Multiple mechanisms for n-3 LCPUFA-mediated attenuation of structural remodeling have been suggested. Activation of the cyclic guanine monophosphate/protein kinase G pathway, thereby reducing nitric oxide synthase levels and Smad2/3-mediated fibrosis, has been proposed [22]. In addition, downregulation of key upstream remodeling signaling molecules such as protein kinase B, p38, extracellular signal related kinase, and/or epidermal growth factor has been reported [19, 21].

A reasonably consistent finding has been that in order for n-3 LCPUFAs to be effective, they must be given before remodeling becomes established. The simultaneous atrial and ventricular pacing model (SAVP), which causes atrial structural remodeling and AF inducibility, has been used to show that prophylactic supplementation with n-3 LCPUFAs is more effective at reducing AF than n-3 LCPUFAs started after remodeling has begun. SAVP in dogs causes atrial inflammation, dilatation, and cellular hypertrophy [25, 26]. After 14 days of SAVP there is significant atrial scarring and AF inducibility. Fibrosis of cardiac tissue is usually the last stage in the structural remodeling cascade and is generally irreversible [27, 28]. Supplementing dogs with n-3 LCPUFAs (850 mg/day EPA+DHA) beginning 7 days after SAVP began (“postinjury n-3 LCPUFAs”) significantly increased EPA+DHA levels in plasma and atrial tissue, but did not prevent AF inducibility compared to dogs that were paced, but did not receive n-3 LCPUFAs (inducibility in postinjury n-3 LCPUFA group 104% of no n-3 LCPUFA group [*P* = NS]). Similarly, atrial fibrosis was not attenuated by postinjury n-3 LCPUFAs (postinjury n-3 LCPUFA collagen area fraction 95% of no n-3 LCPUFA group [*P* = NS]) [26]. However, when n-3 LCPUFAs are given before SAVP is begun (starting 7 days before pacing and continuing through 14 days of pacing), both atrial fibrosis and AF inducibility are significantly reduced. This “prophylactic” supplementation with n-3 LCPUFAs reduces AF inducibility by 73% (*P* < 0.01) and atrial fibrosis by 43% (*P* < 0.05) compared to dogs paced, but not given n-3 LCPUFAs [25]. These data suggest that n-3 LCPUFAs given after the activation of the structural remodeling response are less effective in treating the resulting disease.

N-3 LCPUFAs exhibit similar effects to other known antiremodeling agents such as statins, angiotensin converting enzyme inhibitors (ACEi), and angiotensin receptor blockers (ARBs). The rapid ventricular pacing model (RVP) has been developed to mimic congestive heart failure (CHF) [29]. Several weeks of RVP in dogs cause ventricular dilatation and dysfunction, in addition to atrial fibrosis and AF inducibility [29, 30]. This model has been used to test the effects of ACEi, ARBs, and statins, as well as n-3 LCPUFAs in the setting of heart failure. Dogs subjected to 5 weeks of RVP, but given prophylactic n-3 LCPUFAs (5.28 g/day EPA+DHA starting 2 weeks before pacing began), had a 67% reduction in AF duration (*P* < 0.05) and a 70% decrease in atrial fibrosis (*P* < 0.01) compared to unsupplemented RVP dogs [19]. Enalapril and simvastatin have similar effects in the same model [31, 32]. Although no study has demonstrated that n-3 LCPUFAs and ACEi or statins work via the same mechanism, they do produce similar effects in the setting of structural remodeling induced disease. It may be possible that the effects of n-3 LCPUFAs are overshadowed by the similar effects of modern clinical agents when they are used together.

## 4. Do Clinical Trials Demonstrate an Antiremodeling Effect of n-3 LCPUFAs?

Recent clinical trials are consistent with the antiremodeling hypothesis of n-3 LCPUFA action. The positive GISSI Prevenzione trial mentioned above randomized patients several days or weeks post-MI, at a time when cardiac tissue was likely undergoing remodeling, and in an era before widespread use of pharmacological/surgical antiremodeling therapy for secondary prevention. A 2008 followup to GISSI Prevenzione involving 7,000 patients, called GISSI-HF, showed a significant ~9% reduction in the risk of a major cardiovascular event (SCD, MI, stroke, and hospitalization for CVD) in heart failure patients taking n-3 LCPUFAs (death or admission to hospital for CVD reason relative risk 0.92, total mortality relative risk 0.91) [33]. It is notable that ~60% of patients had only mild heart failure at baseline (New York Heart Association [NYHA] Class II). The fact that n-3 LCPUFAs were administered early in the development of heart failure may have been related to the positive result. This idea is supported by the findings of a smaller study involving 133 patients with mild heart failure at baseline (NYHA Class I or II) [34]. These patients were followed for 12 months and the group randomized to receive n-3 LCPUFAs saw a 10% improvement in LV ejection fraction and more than 25% transitioned to a lower NYHA class. Data from 2 observational studies further support a beneficial effect of prophylactic n-3 LCPUFAs in heart failure. The 2004 Cardiovascular Health Study (CHS) used a cohort of ~5,000 subjects who were in their 70's at baseline (mean age 72.8) and free of heart failure, followed them for 12 years, and reported a heart failure hazard ratio of 0.68 for those who ate fish ≥5 times/week compared to those only eating fish ≤1 time/month [35]. The Japan Collaborative Cohort Study for Evaluation of Cancer Risk (JACC) study looked at the relationship between fish intake and heart failure in almost 58,000 Japanese subjects free of heart failure at baseline. Over ~13 years of followup there was an inverse association between fish intake and heart failure (hazard ratio 0.58 comparing highest quintile of fish intake to lowest) [36]. 

Similarly, a 2008 post hoc analysis of clinical data by Macchia et al. looked at ~3,000 post-MI patients and found that those prescribed n-3 LCPUFAs within 1 year post-MI had a reduced risk of developing AF (relative risk 0.19 in n-3 LCPUFA group) [37]. Observational studies such as the above-mentioned CHS and the Kuopio Ischemic Heart Disease Risk Factor Study showed that increasing fish (or fish oil) intake was associated with a reduced risk of AF [38, 39]. The CHS reported an AF relative risk of 0.69 for those who ate fish ≥5 times/week compared to those only eating fish ≤1 time/month. The 2009 Kuopio study followed their population (2,174 men) for ~18 years and found an AF hazard ratio of 0.62 for the highest versus lowest quartile of serum DHA. It is notable that in the CHS, the event curves for AF incidence do not start to diverge until after year 6, and the final event rate was ~20% (which is higher than in many modern trials).

Data concerning the effect of n-3 LCPUFAs on AF after cardiac surgery is somewhat more difficult to interpret. There are 6 published clinical trials looking at the ability of n-3 LCPUFAs to prevent postoperative AF; 3 are positive, 2 are negative, and 1 is inconclusive [40–45]. The conflicting results are likely the product of the complex pathophysiology of postoperative arrhythmias as well as differences in the definition of AF used. Patients included in these trials had a wide variety of concomitant conditions including MI, valve disease, heart failure, and so forth. It is notable, however, that both negative trials had in excess of 70% of patients taking *β* blockers, statins, and ACEi, whereas only ~50% of patients were using those drugs in the positive trials. Most of these trials are single-center trials involving ≤200 patients. We await the results of the OPERA trial, a multicenter study aiming to enroll over 1,500 patients, studying perioperative n-3 LCPUFA intake and postoperative AF (clinicaltrials.gov identifier NCT00970489).

## 5. Obtaining Adequate n-3 LCPUFA Intake

The n-3 LCPUFA supplementation protocols used in the studies mentioned above vary dramatically from a few hundred milligrams/day to 6 or more grams/day. Some studies use pure EPA or pure DHA, some use a combination of both. Some studies observe for years or months, others only observe for days. Thus, it can be difficult to infer the ideal dosing regimen which will be effective in preventing structural remodeling. Observational studies correlating fish intakes with CVD have found that eating fatty fish 5 or more times/week results in an EPA+DHA intake of approximately 1 g/day, and significant benefits have been observed with those dosages [46, 47]. Supplementation studies in humans have shown that significant increases in blood phospholipids can be achieved with 0.75–1.5 g/day of n-3 LCPUFAs in 3 weeks with additional, but smaller, increases seen if supplementation continues for 3 more weeks [48]. If supplementation is discontinued, EPA levels (and to a slightly lesser extent, DHA levels) can drop to pre-supplementation values in as little as 2 weeks [49]. As seen from the clinical, observational, and animal studies mentioned above, n-3 LCPUFAs are most effective when given prophylactically. From these data, it appears that protective effects of n-3 LCPUFAs could be seen with relatively low-dose supplementation (1 g/day or possibly less) without interruption for at least several weeks before a cardiovascular event occurs.

These doses are attainable from dietary intake of fish. Those who find fish unacceptable, unpalatable or who worry about methylmercury contamination can acquire adequate intakes from pharmaceutical supplements; although a 2006 meta-analysis concluded that even for women of childbearing age, the benefits of modest fish intake exceeded the potential risks [3]. Subjects with already high levels of n-3 LCPUFA intake could potentially benefit from even greater intake. The positive JACC trial mentioned above involved a large Japanese population where the lowest quintile of fish intake corresponded to a daily n-3 LCPUFA intake of 1 g. The Japanese EPA lipid intervention study (JELIS) was a large trial involving over 19,000 hypercholesterolemic patients (total cholesterol ≥251 mg/dL) published in 2007 [50]. Although the study used a dose of 1.8 g/day EPA in a population that already had a high intake of fish, there was a significant 18% reduction in major coronary events after 5 years of followup. However, more testing would be necessary before the benefits of chronic high n-3 LCPUFA intake could be determined.

Plasma phospholipid levels of EPA+DHA or red blood cell membrane levels of EPA+DHA (the Omega-3 index) have both been found to correlate with incidence of CVD [51, 52]. Both can be calculated from a blood test and the Omega-3 index has been put forward as a potential biomarker of coronary heart disease risk [52].

## 6. Conclusion

n-3 LCPUFAs are probably best considered as multipotent molecules with effects that differ depending on dose, supplementation method, supplementation duration, and disease model studied. Caution should be exercised in translating results from the basic science lab to the clinic. Although n-3 LCPUFAs have antiatherogenic and antithrombotic properties, and alter cardiac electrophysiology, it is plausible that the beneficial effects sometimes observed are due to anti-structural remodeling properties. It may be that at usual oral doses, in the setting of diseases with an active remodeling component, n-3 LCPUFA effects on remodeling are more relevant than other effects. Clinical trials aiming to study the effect of n-3 LCPUFAs on CVD may have the greatest chance of success if n-3 LCPUFAs are given early in the development of a disease where the arrhythmia was related to structural remodeling, and the patients are followed for long enough to observe an antiremodeling effect.

## Figures and Tables

**Table 1:** Randomized controlled trials of n-3 LCPUFAs in CVD.

Study	Subjects	Intervention	Followup	Effect of n-3 LCPUFAs	Rn	Pl	Bl
DART Burr et al. [6]	2033 post-MI men	fish diet	2 years	↓ total mortality (29%) ↔ reinfarction, IHD death			
GISSI Marchioli et al. [8]	11323 post-MI	1.0 g/day n-3 LCPUFAs	1 year	↓ total mortality (RR 0.59), SCD (RR 0.47)	X		X
Alpha-Omega Kromhout et al. [11]	4837 post-MI	0.4 g/day n-3 LCPUFAs	3.5 years	↔ rate of major cardiovascular event (RR 1.01)	X	X	X
OMEGA Rauch et al. [12]	3851 post-MI	1.0 g/day n-3 LCPUFAs	1 year	↔ SCD (OR 0.95), total mortality (OR 1.25)	X	X	X
SOFA Brouwer [18]	546 ICD	2.0 g/day FO	1 year	↔ ICD intervention for VF/VT, all cause mortality (RR 0.86)	X	X	X
Leaf et al. [53]	402 ICD	2.6 g/day n-3 LCPUFAs	1 year	↔ ICD intervention for VF/VT (RR 0.72)	X	X	X
Brouwer et al. [13]	200 ICD	1.3 g/day n-3 LCPUFAs	0-2 years	↔ ICD intervention for VF/VT (RR 1.26)	X	X	X
Kowey et al. [15]	663 AF without structural heart disease	4–8 g/day n-3 LCPUFAs	0.5 years	↔ AF recurrence (HR 1.22)	X	X	X
GISSI-HF Tavazzi et al. [33]	7000 NYHA II–IV	1.0 g/day n-3 LCPUFAs	4 years	↓ time to death or hospital admission for cardiovascular reason (RR 0.92), total mortality (RR 0.91)	X	X	X
Nodari et al. [34]	133 NYHA I-II	1.0 g/day n-3 LCPUFAs	1 year	↑ LV ejection fraction (10%) ↓ NHYA classification	X	X	X
Heidarsdottir et al. [44]	168 CABG/valve repair	1.2 g/day EPA + 1.0 g/day DHA	To discharge	↔ POAF	X	X	X
Calò et al. [40]	160 CABG	2.0 g/day n-3 LCPUFAs	To discharge	↓ POAF (15% versus 33% for controls) ↓ hospital stay ↔ all cause mortality	X		X
Saravanan et al. [5, 42]	108 CABG	2.0 g/day n-3 LCPUFAs	To discharge	↔ POAF	X	X	X
Heidt et al. [45]	102 CABG	100 mg/kg FO	To discharge	↓ POAF (17% versus 31% for controls)	X	X	X
Farquharson et al.[43]	194 CABG/valve repair	4.6 g/day n-3 LCPUFAs	To discharge	↔ POAF ↓ length of stay in ICU	X	X	X
JELIS Yokoyama et al. [50]	18645 hypercholesterolemic	1.8 g/day EPA	5 years	18% ↓ major coronary events ↓ unstable angina, nonfatal coronary events	X	X	X
OPERA Mozaffarian *ongoing *	*∼*1500 open cardiac surgery	10 g/day n-3 LCPUFAs pre-op, 2 g/day n-3 LCPUFAs post-op	To discharge	*Primary completion estimated May 2012*	X	X	X

MI: myocardial infarction, ICD: implantable cardioverter defibrillator, AF: atrial fibrillation, NYHA: New York Heart Association, LV: left ventricle, CABG: coronary artery bypass graft, FO: fish oils, EPA: eicosapentaenoic acid, DHA: docosahexaenoic acid, IHD: ischemic heart disease, RR: relative risk, SCD: sudden cardiac death, OR: odds ratio, VF: ventricular fibrillation, VT: ventricular tachycardia, HR: hazard ratio, POAF: postoperative, ICU: intensive care unit, Rn: randomized, Pl: placebo controlled, Bl: blinded.

**Table 2:** Observational studies of n-3 LCPUFAs in CVD.

Study	Subjects	Followup	Results
Physicians' Health Study Albert et al. [7]	20551 healthy men	11 years	Fish intake on RR of SCD: 1/week = 0.48 Fish intake not associated with MI, non-SCD, and total cardiovascular mortality

Framingham Study Shen et al. [16]	4526 healthy	4 years	RR of AF in quartiles of fish intake: *Q*1 = 1, *Q*2 = 1.11, *Q*3 = 0.92, *Q*4 = 1.18

Women's Health Initiative Berry et al. [17]	44720 healthy postmenopausal women	6 years	RR of AF in quartiles of fish intake: *Q*1 = 1, *Q*2 = 1.3, *Q*3 = 1.23, *Q*4 = 1.17

Rotterdam Study Brouwer et al. [18]	5184 subjects	6.4 years	RR of AF in tertiles of fish intake: *T*1 = 1, *T*2 = 1.22, *T*3 = 1.18

Cardiovascular Health Study Mozaffarian et al. [38]	4815 healthy elderly	12 years	Fish intake on HR of HF 1–4/week = 0.8–0.69 5+/week = 0.68 fish intake on RR of AF: 1–4/week = 0.72 5+/week = 0.69

Japan Collaborative Cohort Study for Evaluation of Cancer Risk Yamagishi et al. [36]	57972 healthy	12.7 years	HR of death from HF in quintiles of fish intake *Q*1 = 1, *Q*2 = 0.69, *Q*3 = 0.56, *Q*4 = 0.60, *Q*5 = 0.58

Kuopio Ischemic Heart Disease Risk Factor Study Virtanen et al. [39]	2174 healthy men	17.7 years	↑ serum DHA associated with ↓ AF RR = 0.62 highest versus lowest quartile

RR: relative risk, SCD: sudden cardiac death, MI: myocardial infarction, AF: atrial fibrillation, HR: hazard ratio, HF: heart failure, DHA: docosahexaenoic acid.
